# Problematic internet and social-media use, stressful life events, depressive symptoms, and suicidal behaviors among university students in Cyprus: a cross-sectional study

**DOI:** 10.1007/s44192-026-00465-w

**Published:** 2026-05-04

**Authors:** Lenos Hatzimilidonis, Maria Karanikola, Nicos Middleton, Sokratis Sokratous

**Affiliations:** 1 Department of Nursing, School of Health Sciences, Cyprus University of Technology, Limassol, Cyprus; 2 Community Mental Health Nursing Services, State Health Services Organization (SHSO), Nicosia, Cyprus

**Keywords:** Problematic internet use, PIU, Problematic social-media use, Depressive symptoms, Suicidal behaviors, Stressful life events, University students

## Abstract

**Background:**

Problematic internet use (PIU) and problematic social-media use have been associated with depressive symptoms and suicidal behaviors among university students, with limited Mediterranean evidence. This study examined their associations with stressful life events, depressive symptoms, and suicidal behaviors.

**Methods:**

A cross-sectional anonymous online survey conducted among undergraduates at the Cyprus University of Technology. Participants completed Internet Addiction Test–20 (IAT-20) to assess PIU risk, Bergen Social Media Addiction Scale (BSMAS) to assess problematic social-media use, Center for Epidemiologic Studies Depression Scale (CES-D) to assess depressive symptoms, Life Events Scale for Students (LESS-36) to assess stressful life events, and Suicidal Behaviors Questionnaire–Revised (SBQ-R) to assess suicidal behaviors. Correlation and multivariable linear regression analyses examined associations with depressive symptoms and suicidal behaviors.

**Results:**

1002 students completed the survey (45% response rate); 67.7% were female. PIU risk was minimal (51.1%), mild (38.6%), and moderate (10.3%). BSMAS and LESS-36 scores correlated with depressive symptoms (*ρ* = 0.47; *ρ* = 0.30) and suicidal behaviors (ρ = 0.24; ρ = 0.31; all *p* < 0.001). Adjusted analyses showed depressive symptoms were associated with female gender, mild–moderate PIU, problematic social-media use, and stressful life events. Suicidal behaviors were associated with male gender, non-Cypriot nationality, family history of mental illness, screen time, mild–moderate PIU, stressful life events, and depressive symptoms.

**Conclusions:**

Problematic internet and social-media use and stressful life events were associated with depressive symptoms and suicidal behaviors; longitudinal research is needed to clarify temporal relationships.

## Introduction

Excessive engagement with internet-based technologies and social-media platforms has emerged as a significant behavioral-health concern among university students, who appear particularly vulnerable to the psychological correlates of problematic digital use [1, 2]. In this study, the term PIU is used to describe maladaptive or dysregulated patterns of internet engagement. Importantly, classifications derived from the Internet Addiction Test–20 (IAT-20) are interpreted as reflecting risk levels of problematic use rather than clinically diagnosed addiction. A growing body of research indicates that heightened digital engagement is associated with depressive symptoms, anxiety, sleep disturbances, loneliness, and suicidal ideation among adolescents and young adults [1, 3, 4]. Systematic reviews and meta-analyses further suggest that problematic social-media use and problematic smartphone use are associated with mental-health burdens across diverse cultural contexts [1, 2, 5, 6].

The Interaction of Person–Affect–Cognition–Execution (I-PACE) model provides a conceptual framework that has been used to contextualize associations between problematic digital behaviors and mental health outcomes across diverse populations [7, 8]. Within this framework, PIU is understood as occurring in conjunction with individual characteristics, affective and cognitive processes, and self-regulatory capacities. Previous research has reported that higher levels of stress, depressive symptoms, and suicidality often co-occur with problematic patterns of digital engagement. In the present study, the I-PACE model is used as an interpretive lens rather than to infer causal mechanisms.

Concerns regarding problematic digital behavior intensified during the COVID-19 pandemic. Prolonged lockdowns increased reliance on digital technologies for academic and social functions, a shift associated with poorer sleep and heightened emotional distress [9–11]. Post-pandemic evidence suggests that problematic digital engagement may co-occur with psychological difficulties and may be involved in bidirectional relationships with emotional distress and maladaptive online behavior [1–3, 12–14].

Despite extensive international research, evidence from Mediterranean and Southern European contexts remains limited. Sociocultural factors such as strong family cohesion, distinctive academic expectations, and culturally shaped interaction patterns may influence both digital behaviors and vulnerability to emotional distress [14–17]. In Cyprus, empirical evidence remains scarce despite high levels of smartphone and social-media use among young people [18].

Although previous international and local studies have examined mental-health outcomes or specific forms of digital behavior in isolation, this focus has limited understanding of how problematic digital engagement, psychosocial stressors, and mental-health indicators co-occur within university populations [17, 19–21]. To address this gap, the present study adopts an integrative, associational approach by examining problematic internet and social-media use, stressful life events, depressive symptoms, and suicidal behaviors within a single analytical framework among university students in a Mediterranean context.

In Cyprus, prior work has examined PIU in relation to general psychopathology among college students [22], broader population mental-health outcomes during the COVID-19 period [23, 24] and descriptive patterns of social-media engagement among young people [18]. In Greece, student research has linked PIU with loneliness and mental-health indicators [25] and has supported validated measurement of problematic social-media use in university populations [26]. However, within Cyprus and the broader Mediterranean student literature, important gaps remain. In particular, relatively little is known about the joint examination of PIU, problematic social-media use, psychosocial stress exposure, depressive symptoms, and suicidal behaviors within a single analytical framework. The present study contributes to this literature by examining these domains jointly in a large university student sample from Cyprus using validated measures (IAT-20, BSMAS, LESS-36, CES-D, SBQ-R) and multivariable analyses within an explicitly cross-sectional, non-causal framework.

Informed by the I-PACE model as a conceptual framework, this study aimed to examine cross-sectional associations among PIU, problematic social-media use, stressful life events, depressive symptoms, and suicidal behaviors among university students in Cyprus. Specifically, the study sought to (1) examine associations among problematic digital behaviors, stressful life events, depressive symptoms, and suicidal behaviors; (2) compare depressive and suicidal outcomes across levels of PIU risk; and (3) examine independent associations with depressive symptoms and suicidal behaviors using multivariable regression analyses within an explicitly cross-sectional and non-causal analytic framework.

## Methods

### Study design

A cross-sectional anonymous online survey was conducted between September 2023 and July 2024 among undergraduate students at the Cyprus University of Technology. Cross-sectional online surveys are widely used in research on problematic digital behavior and mental health in university populations [15, 25, 27]. Reporting of this study was informed by the STROBE guidelines for cross-sectional research. The study was observational and non-interventional in scope.

## Participants

Eligible participants were actively enrolled undergraduate students aged ≥ 18 years with valid university registration and an institutional email account.

## Sampling strategy

An open invitation recruitment strategy was employed, whereby all eligible undergraduate students (*N* = 2,225) were invited to participate via official university email announcements distributed through the institution’s digital platform. A total of 1002 students completed the survey (response rate: 45%). Participation was voluntary and without incentives. Consequently, the analytic sample represents a self-selected subset of the invited student population and should be considered a voluntary response sample rather than a probability-based sample or census. This strategy was intended to maximize coverage of the target population.

## Data collection

Data collection was conducted using a secure institutional online platform. Weekly reminder emails were sent during the first month, followed by biweekly reminders thereafter. However, no reminders were distributed within two weeks of examination periods to minimize potential stress-related response bias. The survey required approximately 10 –12 min to complete. The online questionnaire was configured using mandatory (forced-response) settings for all items. Participants were required to complete each question before proceeding to subsequent sections of the survey, thereby preventing item-level nonresponse. As a result, the final dataset contained no missing values at the item level, and no statistical procedures for handling missing data (e.g., listwise deletion, pairwise deletion, or multiple imputation) were necessary. Although this approach ensured data completeness, forced-response formats may increase the likelihood of response bias, particularly for sensitive or psychologically demanding items [28].

Technical restrictions preventing multiple submissions could not be implemented due to anonymity requirements. Participants were instructed to complete the questionnaire only once, and no response patterns suggestive of duplicate submissions were identified. Overall, the recruitment and data-collection procedures are consistent with established practices in prior digital-behavior and mental-health research [11, 20, 25].

## Ethical safeguards

Upon accessing the survey link, participants reviewed an electronic information sheet describing the study objectives, the voluntary nature of participation, confidentiality safeguards, and the estimated completion time. Electronic informed consent was obtained prior to participation. Participants were informed that they could discontinue participation at any time, without providing a reason and without any academic, personal, or other adverse consequences, should they experience discomfort or distress. The platform was configured not to record email addresses, IP addresses, or device identifiers, thereby ensuring complete anonymity.

Given the inclusion of items assessing emotional distress and suicidal behaviors, the first page of the survey provided information about free and confidential psychological support services available through the Cyprus University of Technology Counseling Center, as well as encouragement to seek professional support if participation elicited distress. Given the study design, real time identification of participants or individualized follow-up was not possible. This approach, including the balance between anonymity and participant safeguarding, was reviewed and approved by the national ethics committee.

Prior to analysis, routine data quality checks were performed, including assessment of internal consistency of scale responses, screening for implausible or out-of-range values, and examination of response patterns to identify potential inconsistencies or duplicate entries.

### Measures

All psychological instruments were administered using validated Greek versions. Appropriate permissions for the use of the instruments were obtained where required.

## Sociodemographic variables

Participants reported gender, age, nationality, academic year, relationship status, living arrangement, employment status, parental education, parental financial status, Academic Performance (GPA), family history of mental illness, and average daily time spent on the internet and social media. These variables were selected based on prior evidence linking sociodemographic characteristics with digital-use patterns and mental-health outcomes [19, 29, 30].

## Internet addiction test (IAT-20)

PIU was assessed using the Internet Addiction Test–20 (IAT-20), originally developed by Young [31] and validated in Greek by Tsimtsiou et al. [32]. The instrument comprises 20 items evaluating characteristic patterns of internet-use behaviors and their impact across multiple functional domains, including compulsive use tendencies, diminished control over use, cognitive and emotional preoccupation, neglect of academic and daily responsibilities, interpersonal difficulties, and mood-related consequences. Items are rated on a five-point Likert scale ranging from 1 (“rarely”) to 5 (“always”), with responses reflecting participants’ experiences during the preceding month. Total scores are calculated by summing item responses, yielding a theoretical range from 20 to 100, with higher scores indicating greater levels of PIU. Established cut-off thresholds were applied to describe PIU risk categories: 20–30 (minimal risk), 31–49 (mild risk), 50–79 (moderate risk), and 80–100 (severe risk). These categories represent gradations of problematic use severity rather than clinical diagnoses of addiction. In the analyses, IAT-20 scores were treated as a categorical variable (minimal vs. mild–moderate PIU risk). Internal consistency in the present sample was satisfactory (Cronbach’s α = 0.79).

### Bergen social media addiction scale (BSMAS)

Problematic social-media use was assessed using the Bergen Social Media Addiction Scale (BSMAS), originally developed by Andreassen et al. [33] and validated in Greek university populations by Dadiotis et al. [26].The instrument comprises six items evaluating characteristic patterns of social-media engagement across core behavioral-addiction dimensions, including salience, tolerance, mood modification, relapse, withdrawal, and conflict. Items are rated on a five-point Likert scale ranging from 1 (“very rarely”) to 5 (“very often”), with responses reflecting participants’ experiences over the preceding 12 months. Total scores are calculated by summing item responses, yielding a theoretical range from 6 to 30, with higher scores indicating greater levels of problematic social-media use. BSMAS scores were treated as continuous variables in all analyses. Internal consistency in the present sample was satisfactory (Cronbach’s α = 0.81).

### Center for epidemiologic studies depression scale (CES-D)

Depressive symptoms were assessed using the 20-item Center for Epidemiologic Studies Depression Scale (CES-D), originally developed by Radloff [34] and previously validated for use in Cypriot university populations [35]. The CES-D evaluates the frequency of depressive symptomatology experienced during the preceding week and captures multiple domains of depressive symptoms, including affective, somatic/behavioral, cognitive, and interpersonal aspects. Items are rated on a four-point Likert scale ranging from 0 (“rarely or none of the time”) to 3 (“most or all of the time”). Positively worded items (Items 4, 8, 12, and 16) were reverse-scored prior to computing total scores. Total CES-D scores were calculated by summing item responses, yielding a theoretical range from 0 to 60, with higher scores indicating greater depressive symptom severity. CES-D scores were treated as continuous variables in all analyses. Internal consistency in the present sample was satisfactory (Cronbach’s α = 0.82).

### Suicidal behaviors questionnaire–revised (SBQ-R)

Suicidal thoughts and behaviors were assessed using the Suicidal Behaviors Questionnaire–Revised (SBQ-R), a four-item self-report instrument validated across clinical and non-clinical populations [36]. Items assess lifetime suicidal ideation and attempts (Item 1; scored 1–4), frequency of suicidal ideation during the previous 12 months (Item 2; scored 1–5), threat of suicide attempt (Item 3; scored 1–3), and perceived likelihood of future suicidal behavior (Item 4; scored 0–6). Total scores were calculated by summing item responses, yielding a theoretical range from 3 to 18, with higher scores indicating greater severity of suicidal phenomena. In the present study, SBQ-R scores were treated as continuous variables. The Greek version used in this study was produced through a forward–backward translation procedure to ensure conceptual equivalence. The translated version was additionally reviewed by two psychiatrists for face validity and pilot-tested with a small group of undergraduate students (*n* = 15) to confirm clarity and comprehensibility of item wording. Internal consistency in the present sample was satisfactory (Cronbach’s α = 0.73).

### Life events scale for students (LESS-36)

Stressful life events occurring during the preceding 12 months were assessed using the Life Events Scale for Students (LESS-36), originally developed by Linden [37] and validated for student populations [38]. The Greek-adapted version has been previously used in Cypriot university research [35]. The instrument comprises 36 life events representing academic, interpersonal, financial, health-related, and family-related stressors. Each life event is assigned a predefined life-change unit (LCU) weight reflecting its relative stress severity, with the highest-weighted event assigned 100 LCU points. Participants indicate whether each event occurred within the specified recall period, and total scores are calculated by summing the corresponding LCU weights. The theoretical minimum score is 0, with higher scores indicating greater cumulative stress exposure. Because LCU values are additive, the scale does not have a fixed upper bound. In the present sample, LESS-36 scores exhibited substantial variability (range: 14–2020), consistent with the additive LCU scoring system. LESS-36 scores were treated as continuous variables in all analyses. Internal consistency in the present sample was satisfactory (Cronbach’s α = 0.75).

### Sample size and power considerations

An a priori power analysis was conducted prior to the start of data collection using G*Power version 3.1 [39] to inform expectations regarding sample size requirements for the planned statistical analyses [39, 40]. This analysis was intended as approximate guidance rather than as a definitive determination of sample adequacy, given the observational nature of the study and the inclusion of multiple covariates. Participant recruitment involved inviting all eligible undergraduate students to participate in a voluntary, anonymous survey.

For correlation analyses, assuming a two-tailed significance level of α = 0.05, statistical power (1 − β) of 0.90, and a small-to-moderate expected effect size (*r* = 0.24), the estimated minimum sample size was *N* = 179 [39, 40].

For the multivariable linear regression analyses examining depressive symptoms and suicidal behaviors, a fixed-model design (R^2^ deviation from zero) was specified. Conservative assumptions were used (α = 0.05, power = 0.90, small overall effect size f^2^ = 0.02) to provide context for anticipated model sensitivity rather than to establish a strict sample size threshold. Accordingly, results are interpreted with emphasis on effect sizes and confidence intervals rather than on dichotomous power-based thresholds.

### Statistical analysis

All statistical analyses were performed using IBM SPSS Statistics 26.0. Quantitative variables were summarized using means and standard deviations (SD) and medians with interquartile ranges (IQR), whereas categorical variables were presented as absolute and relative frequencies. Normality of continuous variables was assessed using the Kolmogorov–Smirnov test.

Because the CES-D and SBQ-R scores were non-normally distributed, comparisons of depressive symptoms and suicidal behaviors across PIU risk categories (minimal vs. mild–moderate) were conducted using the Mann–Whitney U test. Associations among BSMAS, LESS-36, CES-D, and SBQ-R scores were examined using Spearman correlation coefficients (rho).

Two multivariable linear regression models were constructed to identify factors associated with:

### Depressive symptoms (CES-D)

#### Suicidal behaviors (SBQ-R)

Although the SBQ-R comprises ordinal response items, total scores were treated as continuous variables in regression analyses, in line with established epidemiological and mental-health research, to preserve statistical power and enable estimation of independent associations across the full range of symptom severity.

Independent variables in the regression models included all sociodemographic characteristics presented in Table 1. For the suicidal-behavior model, CES-D score was additionally included to adjust for depressive symptomatology.

**Table 1:** Sample’s characteristics

		*N*	%
Gender	Male	324	32.3
	Female	678	67.7
Country of origin	Cyprus	904	90.2
	Greece	63	6.3
	Abroad	35	3.5
Family status of student	Married/in a relationship	453	45.2
	Single	549	54.8
Living alone	No	606	60.5
	Yes	396	39.5
Occupational status	Unemployed	675	67.4
	Full/part time employment	327	32.6
Year of study	1st	287	28.6
	2nd	243	24.3
	3rd	243	24.3
	4th	212	21.2
	5th	14	1.4
	> 5th year	3	0.3
Academic performance	5-6.5	200	20.0
	6.6–8.4	614	61.3
	> 8.5	188	18.8
Family history of mental illness	No	843	84.2
	Yes	158	15.8
How would you describe the financial situation of the parents?	Low income	179	17.9
	Moderate income	749	74.8
	High income	74	7.4
Estimated total average daily time spent on the internet and social Media (hours), Mean (SD), Median (IQR)		4.7 (2.0)	4 (3–6)

Because no participants were classified in the severe PIU risk category and the moderate-risk subgroup was relatively small (10.3%), the mild and moderate categories were combined into a single mild–moderate PIU risk category for inferential analyses (*n* = 490). This approach improves statistical power and is consistent with prior research indicating comparable psychological profiles across mild and moderate levels of PIU.

Due to non-normality of regression residuals, logarithmic transformations were applied to the dependent variables (CES-D and SBQ-R) prior to regression modeling.

Unstandardized regression coefficients (B), standard errors (SE), standardized regression coefficients (β), and two-tailed *p*-values are reported. Regression coefficients refer to models with log-transformed outcome variables.

Statistical significance was set at *p* < 0.05 (two-tailed). Analytical procedures followed established methodological standards used in behavioral-addiction and mental-health research [2, 14, 20, 41].

### Ethical considerations

The study was approved by the Cyprus National Bioethics Committee (Protocol No. ΕΕΒΚ/ΕΠ/2023/12). All procedures complied with the Declaration of Helsinki and the General Data Protection Regulation (GDPR). Participation was voluntary, anonymous, and required electronic informed consent.

## Results

A total of 1002 undergraduate students participated in the study (response rate: 45%). Most participants were female (67.7%) and of Cypriot nationality (90.2%). Participants reported a mean estimated daily screen time of 4.7 h (SD = 2.0).

Based on Internet Addiction Test–20 (IAT-20) classifications, 51.1% of students were categorized as minimal PIU risk (*n* = 512), 38.6% as mild PIU risk (*n* = 387), and 10.3% as moderate PIU risk (*n* = 103). No participants met criteria for the severe PIU risk category (≥ 80), a pattern previously reported in selected university-student samples [5, 30].

Descriptive statistics for all psychometric variables (CES-D, SBQ-R, LESS-36, BSMAS, and IAT-20) are presented in Table 2.

**Table 2:** Descriptive statistics of CES-D, SBQ-R, LESS-36, IAT-20 and BSMAS scales

	Minimum	Maximum	Mean (SD)	Median (IQR)
Suicidal behaviors (SBQ-*R*)	3.0	16.0	4.3 (2.4)	3 (3–5)
Depression scale (CES-D)	0.0	55.0	17.6 (10.5)	16 (10–24)
Problematic social-media use (BSMAS)	6.0	30.0	13.7 (5.0)	13 (10–17)
Life events stress scale (LESS-36)	14.0	2020.0	235.1 (206.2)	190.5 (90–323)
			N	%
PIU risk classification (IAT–20)	Minimal		512	51.1
	Mild		387	38.6
	Moderate		103	10.3
	Severe		0	0.0

*To address objective* 1, Spearman correlation analyses identified significant positive associations among problematic social-media use, stressful life events, depressive symptoms, and suicidal behaviors. Higher BSMAS scores were moderately correlated with depressive symptoms measured by the CES-D (*ρ* = 0.47, *p* < 0.001) and weakly but significantly correlated with suicidal behaviors measured by the SBQ-R (*ρ* = 0.24, *p* < 0.001).

Exposure to stressful life events, assessed using the LESS-36, was positively associated with both depressive symptoms (*ρ* = 0.30, *p* < 0.001) and suicidal behaviors (*ρ* = 0.31, *p* < 0.001). These findings indicate positive associations between problematic social-media use, cumulative stress exposure, and indicators of emotional distress among university students. Correlation coefficients are summarized in Table 3.

**Table 3:** Associations between PIU, depressive symptoms, suicidal behaviors, problematic social media use, and life events stress scale

	Depression scale (CES -D)		Suicidal behaviors (SBQ-R)	
Problematic social-media use (BSMAS)	0.47***		0.24***	
Life events stress scale (LESS-36)	0.30***		0.31***	
PIU risk classification	Mean (SD)	Median (IQR)	Mean (SD)	Median (IQR)
Minimal	14.1 (9.3)	12 (8–19)	3.8 (1.7)	3 (3–3)
Mild–moderate	21.3 (10.5)	20 (13–28)	4.8 (2.8)	3 (3–6)
*p* (Mann-Whitney test)	< 0.001		< 0.001	

*p < 0.05; **p < 0.01; ***p < 0.001

*In relation to objective* 2, comparisons of mental-health outcomes across PIU risk categories demonstrated significant differences, as presented in Table 3. Students classified as mild–moderate PIU risk reported higher depressive symptoms (CES-D: mean = 21.3, SD = 10.5; median = 20, IQR = 13–28) compared with those in the minimal-risk category (mean = 14.1, SD = 9.3; median = 12, IQR = 8–19), with the difference reaching statistical significance (Mann–Whitney U test, *p* < 0.001).

**Fig. 1:**
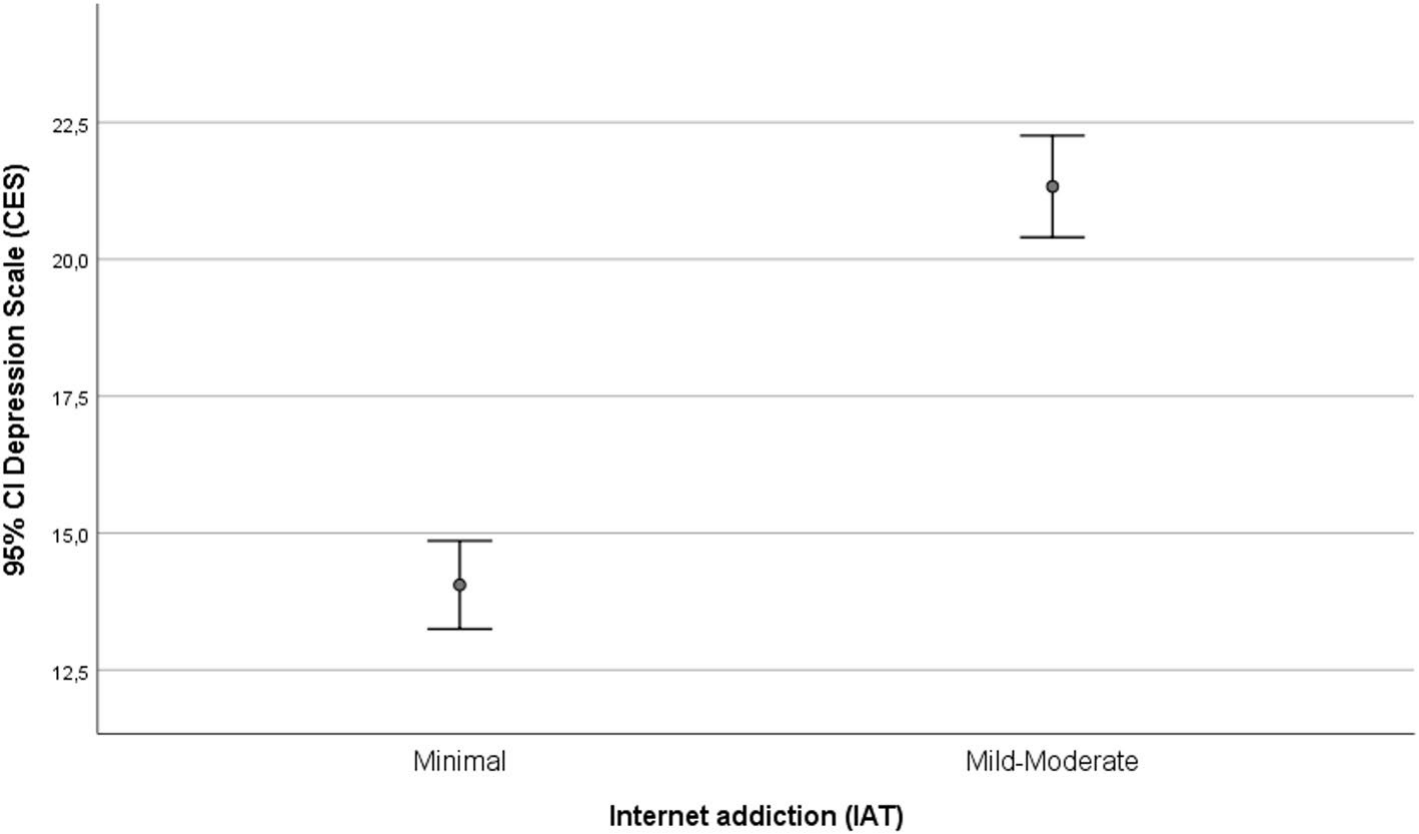
Depressive symptom scores across PIU risk categories

A similar pattern was observed for suicidal behaviors. Students classified in the mild–moderate PIU risk category reported higher SBQ-R scores (mean = 4.8, SD = 2.8; median = 3, IQR = 3–6) compared with those in the minimal PIU risk category (mean = 3.8, SD = 1.7; median = 3, IQR = 3–3), with the difference reaching statistical significance (Mann–Whitney U test, *p* < 0.001). Although median values were identical, distributional differences were observed, including a higher upper quartile in the mild-moderate PIU category, consistent with the significant Mann–Whitney result. These findings indicate higher suicidal-behavior scores among students with mild-moderate PIU risk. Group comparisons are illustrated in Fig. 1.

*Objective* 3 examined adjusted associations between selected demographic, behavioral, and psychosocial variables and depressive symptoms and suicidal behaviors using multivariable linear regression models within a cross-sectional analytic framework.

### Depressive symptoms

Results of the multivariable linear regression model examining factors independently associated with depressive symptoms are presented in Table 4. After adjustment for demographic, academic, behavioral, and psychosocial variables, higher CES-D scores were independently associated with female gender (β = 0.064, *p* = 0.024), mild–moderate PIU-risk classification (β = 0.109, *p* = 0.003), higher BSMAS scores (β = 0.338, *p* < 0.001), and greater exposure to stressful life events (LESS-36; β = 0.195, *p* < 0.001).

**Table 4:** Results of multiple linear regression analysis with dependent variable score on depression scale (CES-D)

	B	SE	β	*p*
Gender (female vs. male)	0.040	0.018	0.064	0.024
Country of origin: Greece versus Cyprus	0.036	0.033	0.030	0.282
Country of origin: Abroad versus Cyprus	0.079	0.044	0.050	0.072
Married/In a relationship (Yes vs. No)	− 0.012	0.017	− 0.021	0.458
Living alone (Yes vs. No)	0.028	0.017	0.047	0.092
Employed (Yes vs. No)	0.006	0.018	0.009	0.756
Year of study	0.001	0.007	0.004	0.901
Academic performance: 6.6–8.4 versus 5-6.5	0.012	0.021	0.021	0.554
Academic performance: >8.5 versus 5-6.5	0.022	0.026	0.029	0.404
Highest level of education of parents: secondary versus primary	0.020	0.065	0.033	0.761
Highest level of education of parents: tertiary versus primary	0.011	0.065	0.018	0.871
Family history of mental illness (Yes vs. No)	0.026	0.022	0.033	0.241
Financial status of parents: moderate-normal income versus low income	− 0.036	0.022	− 0.053	0.098
Financial status of parents: high-income versus low-income	− 0.050	0.036	− 0.044	0.168
Estimated total average daily time spent on the internet and social media	0.005	0.004	0.034	0.242
Mild-moderate PIU risk versus Minimal PIU risk (IAT-20)	0.063	0.021	0.109	0.003
Problematic social-media use (BSMAS)	0.020	0.002	0.338	< 0.001
Total stress event score (LESS-36)	0.001	0.001	0.195	< 0.001

B = unstandardized regression coefficient; SE = standard error; β = standardized regression coefficient. Logarithmic transformation of the dependent variable was used for this analysis

Among the included variables, problematic social-media use (BSMAS) exhibited the largest standardized regression coefficient, followed by stressful life events and PIU risk. All reported associations represent adjusted relationships conditional on the other variables included in the model.

### Suicidal behaviors

The multivariable regression model examining factors associated with suicidal behaviors is presented in Table 5. Higher SBQ-R scores were independently associated with male gender (reference: female; β = − 0.061, *p* = 0.027), non-Cypriot nationality (abroad vs. Cyprus; β = 0.124, *p* < 0.001), family history of mental illness (β = 0.161, *p* < 0.001), lower academic year (β = −0.061, *p* = 0.025), greater daily screen time (β = 0.066, *p* = 0.018), mild–moderate PIU risk category (β = 0.068, *p* = 0.049), greater exposure to stressful life events (LESS-36; β = 0.150, *p* < 0.001), and higher depressive symptom scores (CES-D; β = 0.384, *p* < 0.001).

**Table 5:** Results of multiple linear regression analysis with dependent variable score about suicidal behaviors (SBQ-R)

	B	SE	β	*P*
Gender (female vs. male)	− 0.019	0.009	-0.061	0.027
Country of origin: Greece versus Cyprus	0.029	0.016	0.047	0.077
Country of origin: Abroad versus Cyprus	0.101	0.022	0.124	< 0.001
Married/In a relationship (Yes vs. No)	0.007	0.008	0.023	0.396
Living alone (Yes vs. No)	− 0.006	0.008	− 0.019	0.482
Employed (Yes vs. No)	− 0.005	0.009	− 0.016	0.570
Year of study	− 0.008	0.003	− 0.061	0.025
Academic performance: 6.6–8.4 versus 5-6.5	0.005	0.010	0.017	0.616
Academic performance: >8.5 versus 5-6.5	0.020	0.013	0.052	0.121
Highest level of education of parents: secondary versus primary	− 0.007	0.032	− 0.022	0.833
Highest level of education of parents: tertiary versus primary	− 0.002	0.032	− 0.008	0.938
Family history of mental illness (Yes vs. No)	0.066	0.011	0.161	< 0.001
Financial status of parents: moderate-normal income versus low income	− 0.014	0.011	− 0.040	0.202
Financial status of parents: high-income versus low-income	− 0.019	0.018	− 0.033	0.286
Estimated total average daily time spent on the internet and social media	0.005	0.002	0.066	0.018
Mild-moderate PIU risk versus minimal PIU risk (IAT-20)	0.020	0.010	0.068	0.049
Problematic social-media use (BSMAS)	0.001	0.001	0.005	0.879
Total stress event score (LESS-36)	0.001	0.001	0.150	< 0.001
Depression scale (CES-D)	0.005	0.001	0.384	< 0.001

B = unstandardized regression coefficient; SE = standard error; β = standardized regression coefficient. Logarithmic transformation of the dependent variable was used for this analysis

CES-D scores exhibited the largest standardized regression coefficient in the suicidal-behavior model, followed by family history of mental illness and exposure to stressful life events. All associations reflect adjusted relationships within the cross-sectional analytic framework.

## Discussion

This study examined how PIU and problematic social-media use, stressful life events, depressive symptoms, and suicidal behaviors are interrelated among university students in Cyprus. Overall, the observed associations were consistent with international evidence reporting associations between problematic internet and social-media use, cumulative stress exposure, and higher depressive symptoms and suicidal risk [1, 2, 13, 30]. These patterns accord with studies demonstrating that problematic smartphone and social-media use among young people have been associated with emotional dysregulation, disturbed sleep, anxiety, and loneliness [2, 5, 6, 16]. To our knowledge, relatively limited evidence from Mediterranean university populations has simultaneously examined PIU, problematic social-media use, cumulative stress exposure, depressive symptoms, and suicidal behaviors within the same multivariable framework, allowing the present study to examine their relative and independent associations in a sociocultural specific context.

When interpreted in the light of the I-PACE framework, these associations may be viewed as consistent with interactions between stress-related affective states, maladaptive coping through digital platforms, and diminished regulatory control [7, 8].

Consistent with prior studies from Cyprus and Greece that have PIU use or social-media engagement separately, the present study extends this work by examining these behaviors alongside stressful life events, depressive symptoms, and suicidal behaviors within a single analytical framework [18, 22, 25, 26]. This integrative approach allows for the estimation of independent associations among these domains within a Mediterranean university student population, while accounting for co-occurring stress exposure and depressive symptomatology.

Problematic social-media use was moderately associated with depressive symptoms and weakly associated with suicidal behaviors. Although smaller in magnitude, this association with suicidality is consistent with previous research reporting associations between intensive or dysregulated social-media engagement and indicators of emotional distress. Evidence synthesized in systematic reviews and meta-analyses indicates that problematic social media use is frequently observed alongside emotional instability, increased social comparison, and reduced offline social engagement, highlighting its relevance as an emerging mental-health concern across diverse populations [2, 5, 12, 29].

Stressful life events were also significantly associated with depressive symptoms, consistent with the stress vulnerability framework of emotional distress. University students are frequently exposed to academic pressure, interpersonal conflicts, and financial difficulties, which have been associated with increased psychological burden during late adolescence and early adulthood. The association observed here mirrors previous findings among Cypriot university students using the same LESS-36 measure [35, 42].

Gender patterns partially corresponded with established literature: female students reported higher depressive symptoms [24, 35] whereas male students exhibited higher suicidal-behavior scores. Although large meta-analyses typically find that non-fatal suicidal thoughts and attempts are more common among females, men consistently exhibit higher suicide mortality, which may reflect riskier suicidal behaviors [43]. Non-Cypriot nationality and a family history of mental illness were also associated with higher suicidal-behavior scores, in line with cross-cultural evidence linking adjustment stressors and inherited vulnerability to poorer emotional outcomes [1, 12, 20, 27]. In Mediterranean sociocultural contexts, norms emphasizing emotional restraint and self-reliance among men may discourage disclosure of depressive symptoms while contributing to higher engagement in externalizing or high-risk behaviors, including more severe suicidal actions.

Students classified in the mild–moderate PIU risk category reported higher depressive and suicidal symptoms than those at minimal risk. This pattern is consistent with earlier research documenting graded associations between PIU and psychological distress [13, 19, 30, 44].

Importantly, IAT-20 classifications indicate levels of PIU risk based on a screening instrument and should not be interpreted as clinical diagnoses of internet addiction. Therefore, the findings should be interpreted as reflecting associations with elevated risk of PIU within a non-clinical student sample.

Regression analyses further showed that depressive symptoms were independently associated with gender, PIU, problematic social-media use, and stress exposure, consistent with prior observations that PIU and psychosocial stress are frequently observed alongside emotional difficulties [15, 30]. Suicidal behaviors were independently associated with demographic characteristics, screen time, PIU risk, stress exposure, and depressive symptoms. These findings are consistent with a multidimensional understanding of suicidal risk, shaped by emotional vulnerability, behavioral patterns, and environmental stressors [4, 13, 19, 44].

Given the cross-sectional and non-experimental design of the study, the findings should be interpreted as indicating associations rather than explanatory or causal relationships. The results allow examination of how these variables co-occur within this student population but do not establish the directionality or underlying mechanisms of these relationships.

### Interpretation in the Mediterranean context

These findings offer new insights from a Mediterranean sociocultural context, where strong family ties, collectivistic social norms, and high digital penetration may shape students’ experiences of digital use and emotional well-being. Existing studies in Greece have shown that PIU, loneliness, and intensive social-media engagement are associated with depressive symptoms and reduced well-being among young people [10, 25, 26]. National findings from Cyprus additionally document substantial levels of problematic social-media use and PIU, particularly among adolescents and young adults [14, 18, 22, 23] underscoring the relevance of the present results.

The robust association between stressful life events and depressive symptoms mirrors earlier Cypriot findings using the LESS-36 [35, 42]. The stability of these associations across Mediterranean samples suggests that digital-behavior vulnerabilities may be broadly cross-cultural, while still shaped by regional expectations surrounding academic performance, family obligations, and social connectedness.

#### Public health implications

International literature highlights the role of educational settings in supporting student well-being through digital-well-being and social-media literacy initiatives [45, 46] alongside stress-management and resilience-building programs that strengthen students’ coping resources [46]. Future research could examine the feasibility, acceptability, and ethical considerations of screening approaches for depressive symptoms and PIU within university settings, particularly among groups considered to be at higher vulnerability [21, 47].

Interventions that promote balanced and intentional technology use should be embedded within broader mental-health promotion strategies, given that problematic digital engagement frequently co-occurs with sleep disturbances, emotional dysregulation, and academic difficulties [4, 11, 16, 21]. When considered alongside national findings from Cyprus [14, 18, 22, 23] the present results may help inform future research agendas and contribute to wider scholarly discussions on student mental health across diverse sociocultural contexts.

#### Strengths and limitations

This study has several notable strengths. It benefits from a large sample size and the use of five validated psychometric instruments (IAT-20, BSMAS, CES-D, SBQ-R, and LESS-36), which enhances measurement reliability. The joint examination of PIU, problematic social-media use, stressful life events, and sociodemographic variables provides a comprehensive framework for understanding mental-health vulnerability among university students. The application of multivariable analytical models further strengthens the evaluation of independent associations within this population.

Several limitations should be acknowledged. First, the cross-sectional design precludes conclusions regarding temporal ordering or causality, a limitation inherent to observational research and widely recognized in studies of digital behavior and mental health [11, 14, 18, 25, 44]. Second, all variables were assessed using self-reported measures, which may be subject to recall or social-desirability bias. Third, the sample was drawn from a single academic institution, which may limit generalizability to other university populations in Cyprus or the wider Mediterranean region.

In addition, the response rate (45%) may limit the representativeness of the sample and the generalizability of the findings. Although this rate is comparable to other voluntary online surveys conducted among university populations, participation was based on self-selection, introducing the potential for self-selection bias, as respondents may differ systematically from nonparticipants. Consequently, the findings should be interpreted as reflecting associations within the participating sample rather than population-level estimates. Fourth, although the survey was administered anonymously, which precluded technical enforcement of single-entry restrictions, participants were instructed to complete the questionnaire only once, and no response patterns indicative of duplicate submissions were identified. Finally, the study did not incorporate objective digital-behavior metrics (e.g., smartphone activity logs), which recent research suggests may enhance the precision of digital-use assessment [16, 21, 46].

Future research should consider longitudinal and cross-cultural designs to better clarify directionality and contextual influences. Incorporating digital-trace data and exploring potential psychosocial mediators such as sleep quality, emotion regulation, fear of missing out (FOMO), and psychotic-like experiences may further enrich understanding of how PIU relates to depressive and suicidal symptoms [21, 44, 46].

## Conclusions

This study identified significant associations between problematic internet and social-media use, exposure to stressful life events, and higher levels of depressive and suicidal symptoms among university students in Cyprus. The findings underscore the multifactorial nature of emotional vulnerability in young adults, with digital-use patterns, psychosocial stress burden, gender, and depressive symptoms each demonstrating independent associations with mental-health outcomes.

When interpreted within the I-PACE model as a conceptual and interpretive framework, rather than as an explanatory mechanism, the results suggest that PIU may co-occur with stress-related affective states and difficulties in self-regulation. These patterns are consistent with broader literature describing the frequent co-occurrence of PIU, problematic social-media use, emotional distress, and psychosocial stress exposure in university populations.

From a population mental-health perspective, the findings add to a growing body of evidence suggesting that PIU and problematic social-media use may represent an area of concern among young adults. They further highlight the value of continued research on patterns of internet and social-media use and psychosocial stressors in university settings, particularly within Mediterranean sociocultural contexts that remain underrepresented in the literature. Future studies employing longitudinal designs and objective digital-use measures are needed to clarify temporal relationships and inform the development of ethically sound prevention and mental-health promotion strategies.

## Data Availability

The datasets generated and/or analyzed during the current study are not publicly available due to institutional data protection restrictions. Data are available from the corresponding author upon reasonable request.
